# Jujube leaf green tea extracts inhibits hepatocellular carcinoma cells by activating AMPK

**DOI:** 10.18632/oncotarget.22821

**Published:** 2017-11-30

**Authors:** HX Liu, MQ Xu, SP Li, S Tian, MX Guo, JY Qi, CJ He, XS Zhao

**Affiliations:** ^1^ Jujube Scientific Research and Applied Center, Life Science College, Luoyang Normal University, Luoyang, China

**Keywords:** jujube leaf green tea extracts, hepatocellular carcinoma, AMPK signaling

## Abstract

Here we evaluated the anti-hepatocellular carcinoma activity by the Jujube leaf green tea extracts (JLGTE). We showed that JLGTE exerted anti-proliferative, cytotoxic and pro-apoptotic activities against HepG2 and primary human hepatocellular carcinoma cells. It was however non-cytotoxic to the normal hepatocytes. JLGTE activated AMP-activated protein kinase (AMPK) signaling, which was required for its cytotoxicity against hepatocellular carcinoma cells. Silence of AMPKα1, via targeted short hairpin RNAs or CRISPR-Cas9 genome editing, inhibited JLGTE-induced AMPK activation and HepG2 cell apoptosis. Further, in-activation of AMPK by a dominant negative AMPKα1 (T172A) also alleviated JLGTE's cytotoxicity against HepG2 cells. On the other hand, forced-activation of AMPK by introduction of a constitutively-active AMPKα1 (T172D) mimicked JLGTE's actions and led to HepG2 cell apoptosis. These results suggest that JLGTE inhibits human hepatocellular carcinoma cells possibly via activating AMPK.

## INTRODUCTION

Traditional Chinese Medicines (TCMs) and Chinese herbal compounds have been extensively tested for their anti-cancer abilities [1–3]. Some of the TCMs displayed promising anti-hepatocellular carcinoma (HCC) actions [1–3]. Jujube leaf green tea has been long utilized in ancient China for efficient treatment of several diseases [4]. The current study tested the potential anti-HCC activity by the Jujube leaf green tea extracts (JLGTE). The underlying signaling mechanisms were also tested.

AMP-activated protein kinase (AMPK) is vital in maintaining the energy metabolic balance and homeostasis of glucose and fat metabolism [5, 6]. Recent cancer studies have proposed an important anti-cancer activity by activated AMPK [7–9]. Activation of AMPK could inhibit human cancer cells via different mechanisms. For instance, it has been shown that AMPK could directly associate and activate p53 [10, 11], the latter is a key tumor-suppressor and apoptosis-inducer [12]. Further, activated AMPK could inhibit mTORC1 (mammalian target of rapamycin complex 1, a vital tumor-promoting signaling [13]) via directly inhibiting tuberous sclerosis complex 2 (TSC2) [14, 15]. AMPK-mediated mTORC1 inhibition could also be due to direct inhibition on Raptor [16, 17]. Additionally, studies found that AMPK is key upstream kinase to promote cell autophagic death [18, 19]. AMPK-mediated mTORC1 inhibition and/or Ulk1 activation could sever as the trigger of cell autophagy [18, 19]. A number of anti-cancer agents were shown to inhibit human cancer cells via activating AMPK signaling [8, 20–22]. Meanwhile, forced-activation of AMPK via genetic methods can efficiently inhibit human cancer cells [23–26]. The results of this study indicate that JLGTE inhibits human HCC cells via activating AMPK signaling.

## RESULTS

### Jujube leaf green tea extracts (JLGTE) inhibits human HCC cell survival and proliferation *in vitro*

**Figure 1:**
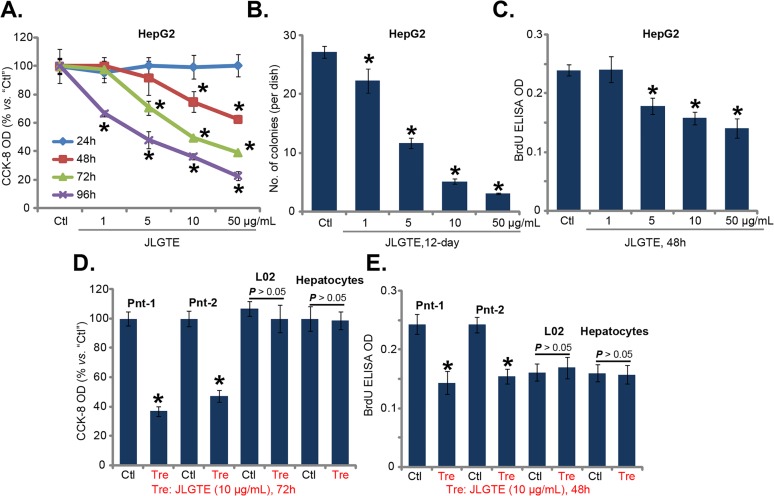
Jujube leaf green tea extracts (JLGTE) inhibits human HCC cell survival and proliferation *in vitro* HepG2 cells **(A-C)**, the primary human HCC cells (Two lines, “Pnt-1/-2”) **(D-E)**, as well as L02 hepatocytes (“L02”) and the primary human hepatocytes (“Hepatocytes”) (D-E) were either left untreated (“Ctl”) or treated with indicated concentration of Jujube leaf green tea extracts (JLGTE), cell survival was assayed by the CCK-8 method (A and D) and colony formation assay (B, for HepG2 cells). Cell proliferation was tested via the BrdU ELISA assay (C and E). Data were presented as mean ± standard deviation (SD, with n=5). ^*^ ***P*** < 0.05 *vs.* “Ctl” group. Experiments in this figure were repeated three times, and similar results were obtained.

This study was designed to test the potential effect of Jujube leaf green tea extracts (JLGTE) on human HCC cells. HepG2 is a well-established human HCC cell line [27, 28]. HepG2 cells were cultured in the complete medium and were treated with various concentrations of JLGTE, from 1-50 μg/mL. Cells were then cultured in the JLGTE-containing medium for applied time, and cell survival was tested by the routine cell counting kit (CCK)-8 assay. Quantified results in Figure 1A demonstrated that JLGTE inhibited HepG2 cell survival in dose- and time-dependent manners. The IC-50 of JLGTE, or the concentration that led to 50% reduction of CCK-8 OD, was close to 10 μg/mL at 72 hours (Figure 1A). It would require at least 48 hours for JLGTE to exert a significant anti-survival effect (Figure 1A). The quantified colony formation assay results in Figure 1B demonstrated that JLGTE treatment significantly decreased the number of viable HepG2 cell colonies (Figure 1B). JLGTE-mediated inhibition on HepG2 colony formation was again dose-dependent (Figure 1B). BrdU incorporation ELISA assay was performed to test cell proliferation. As shown in Figure 1C, treatment with JLGTE dose-dependent decreased BrdU ELISA OD in HepG2 cells, indicating proliferation inhibition.

Next, we tested the effect of JLGTE in the primary human HCC cells. Two lines of primary human HCC cells (“Pnt-1/2”) were provided by Dr. Zhang [29]. The primary cancer cells were treated with JLGTE. CCK-8 assay results in Figure 1D demonstrated that JLGTE (10 μg/mL, 72 hours) significantly inhibited survival of the primary HCC cells. Furthermore, the BrdU ELISA OD of primary HCC cells was also decreased following JLGTE (10 μg/mL, 48 hours) treatment (Figure 1E), suggesting proliferation inhibition. Notably, in the L02 hepatocytes [30] and primary human hepatocytes (provided by Dr. Fan [31]), the very same JLGTE treatment (10 μg/mL, 48/72 hours) failed to inhibit cell survival (Figure 1D) and proliferation (Figure 1E). These results together show that JLGTE selectively inhibits human HCC cell survival and proliferation.

### JLGTE induces apoptosis activation in human HCC cells *in vitro*

**Figure 2:**
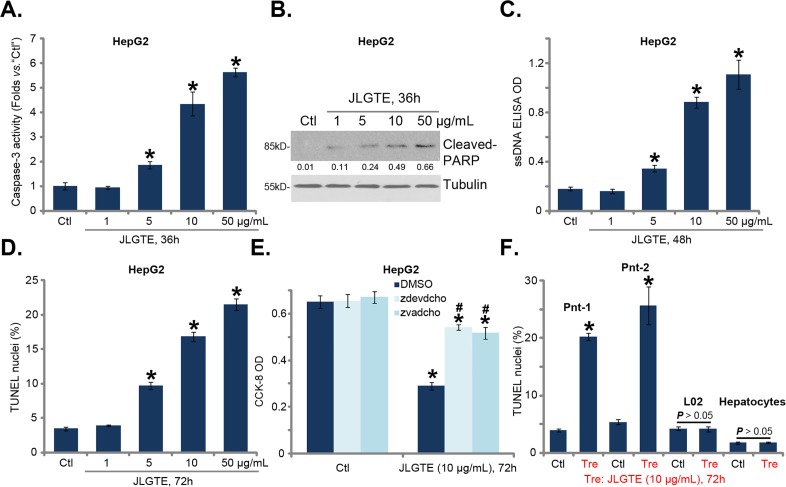
JLGTE induces apoptosis activation in human HCC cells *in vitro* HepG2 cells **(A-E)**, the primary human HCC cells (Two lines, “Pnt-1/-2”) **(F)**, as well as L02 hepatocytes (“L02”) and the primary human hepatocytes (“Hepatocytes”) (F) were either left untreated (“Ctl”) or treated with indicated concentration of Jujube leaf green tea extracts (JLGTE), cell apoptosis was tested by the assays mentioned in the text (A-D, and F); For testing cell survival, cells were also co-treated with the caspase 3 inhibitor z-DEVD-cho (40 μM) or the pan caspase inhibitor z-VAD-cho (40 μM) (E, CCK-8 assay). Cleaved-PARP expression was normalized to the loading control (“Tubulin”) (B). Data were presented as mean ± standard deviation (SD, with n=5). ^*^ ***P*** < 0.05 *vs.* “Ctl” group. ^#^ ***P*** < 0.05 *vs.* “DMSO” (0.1%) (E). Experiments in this figure were repeated three times, and similar results were obtained.

The potential effect of JLGTE on HCC cell apoptosis was analyzed. As shown in Figure 2A, treatment with JLGTE dose-dependently increased the activity of caspase-3 in HepG2 cells. Further, JLGTE induced cleavage of PARP (poly (ADP-ribose) polymerase), the main target of activated-caspase-3 [32, 33] (Figure 2B). JLGTE's effect on PARP cleavage was also dose-dependent (see quantification in Figure 2B). Subsequently, the content of intracellular single strand DNA (“ssDNA”) was also significantly increased in JLGTE-treated HepG2 cells (Figure 2C) [29]. Additionally, the percentage of HepG2 cells with TUNLE-positive nuclei, the well-known indicator of cell apoptosis, was increased following JLGTE treatment (Figure 2D). These results clearly indicated that JLGTE induced apoptosis activation in HepG2 cells.

To study the association between JLGTE-induced apoptosis activation and cytotoxicity, caspase-based apoptosis inhibitors were applied. CCK-8 assay results in Figure 1E showed that z-DEVD-cho (the caspase-3 specific inhibitor) and z-VAD-cho (the pan caspase inhibitor) both inhibited JLGTE (10 μg/mL, 72 hours)-induced viability reduction in HepG2 cells. The results suggested that apoptosis activation mediated JLGTE-induced cytotoxicity against HepG2 cells. Quantified TUNEL staining assay results in Figure 2F demonstrated that JLGTE (10 μg/mL, 48 hours) also induced apoptosis activation in both lines of the primary human HCC cells (“Pnt-1/2”). On the other hand, no TUNEL percentage increase was observed in the JLGTE-treated L02 hepatocytes and primary human hepatocytes (Figure 2F). These results again implied a unique activity of JLGTE against the cancerous cells.

### JLGTE activates AMPK in HCC cells

**Figure 3:**
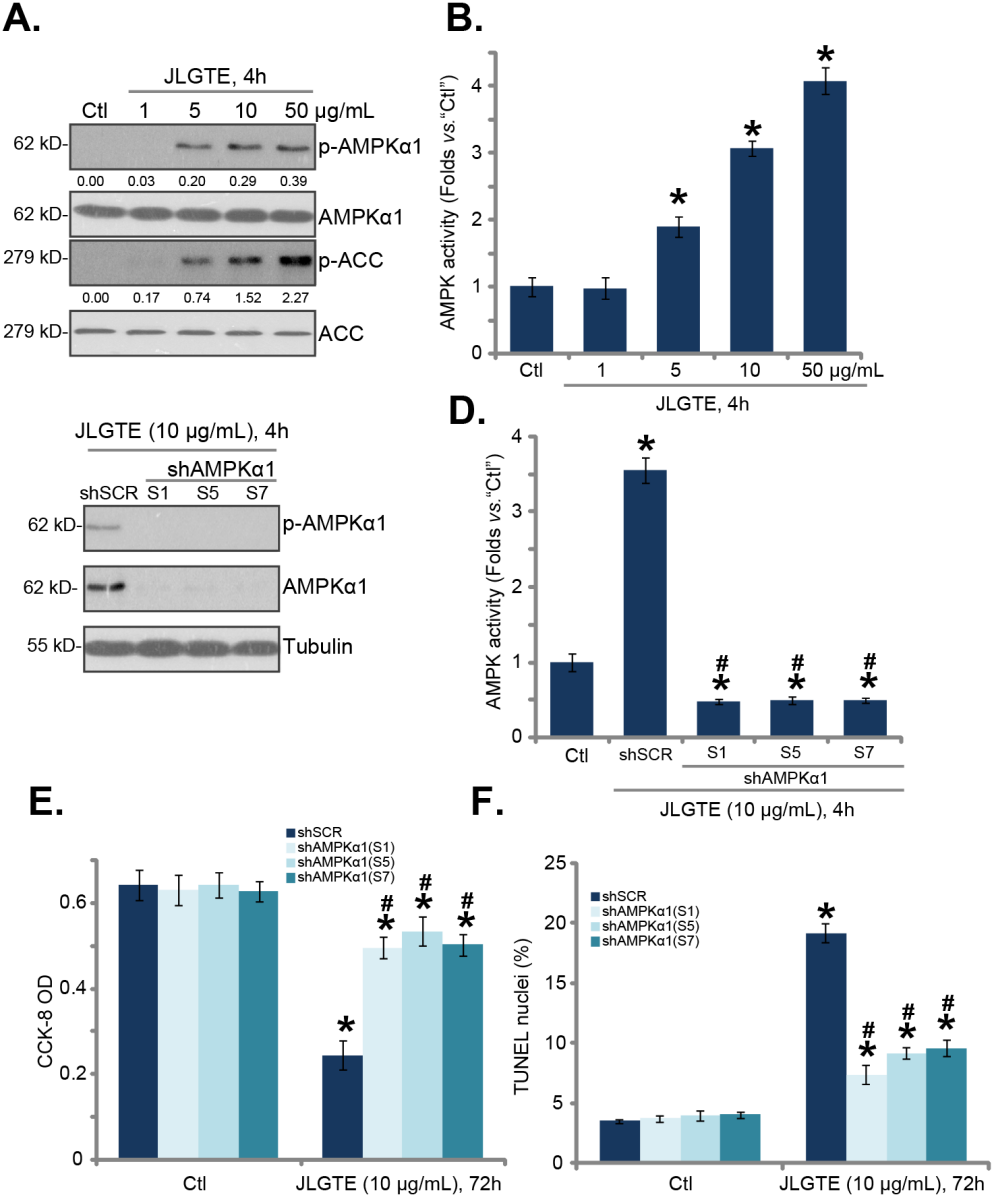
JLGTE activates AMPK in HCC cells HepG2 cells were either left untreated (“Ctl”) or treated with indicated concentration of Jujube leaf green tea extracts (JLGTE), expressions of listed proteins in total cell lysates were shown **(A)**; relative AMPK activity was also tested **(B)**. Puromycin-selected stable HepG2 cells, with scramble control shRNA (“shSCR”) or the listed AMPKα1 shRNA (S1, S5 or S7) were either left untreated (“Ctl”) or treated with JLGTE (10 μg/mL) for the applied time period, AMPKα1 expression **(C)**, AMPK activity **(D)**, cell survival (CCK-8 assay, **E**) and apoptosis (TUNEL staining assay, **F**) were tested. AMPKα1/ACC phosphorylations were quantified (A). Data were presented as mean ± standard deviation (SD, with n=5). ^*^ ***P*** < 0.05 *vs.* “Ctl” group. ^#^ ***P*** < 0.05 *vs.* “shSCR” cells. Experiments in this figure were repeated three times, and similar results were obtained.

As discussed, activation of AMPK has proven to be a fine strategy to inhibit human cancer cells [7–9]. A number of anti-cancer agents were shown to inhibit human cancer cells via activating AMPK signaling [8, 20, 22, 34]. We thus wanted to know the potential effect of JLGTE on AMPK in HCC cells. As shown in Figure 3A, treatment with JLGTE dose-dependently increased phosphorylation of AMPKα1 at Thr-172, which is a key site for AMPK activation [5, 35, 36]. Consequently, acetyl coenzyme A carboxylase (ACC) phosphorylation, the primary downstream target protein of AMPK [5, 35, 36], was also significantly increased by JLGTE (Figure 3A). Expressions of AMPKα1 and ACC were unchanged by the JLGTE treatment (Figure 3A). When analyzing AMPK activity, we found that JLGTE dose-dependently increased AMPK activity in HepG2 cells (Figure 3B). These results implied that JLGTE induced AMPK activation in HepG2 cells.

Whether AMPK activation caused JLGTE-induced cytotoxicity was analyzed next. We utilized short hairpin RNA (shRNA) method. A total of eight different lentiviral AMPKα1 shRNAs, targeting non-overlapping sequences, were designed and added to HepG2 cells. Of these tested shRNAs, Sequence 1 (“S1”), S5 and S7 shRNAs efficiently downregulated AMPKα1 in HepG2 cells (Figure 3C). Consequently, JLGTE (10 μg/mL, 4 hours)-induced AMPKα1 phosphorylation (at Thr-172) (Figure 3C) and AMPK activity increase (Figure 3D) were almost completely blocked by the shRNAs. More importantly, JLGTE (10 μg/mL)-induced HepG2 cell viability reduction (CCK-8 assay, Figure 3E) and apoptosis (TUNEL assay, Figure 3F) were also dramatically inhibited by the AMPKα1 shRNAs. Treatment with the AMPKα1 shRNAs alone didn't change cell survival nor apoptosis (Figure 3E and 3F). These experimental results indicated that activation of AMPK is required for JLGTE-mediated cytotoxicity against HepG2 cells.

### AMPKα1 knockout or dominant negative mutation attenuates JLGTE-induced cytotoxicity in HepG2 cells

**Figure 4:**
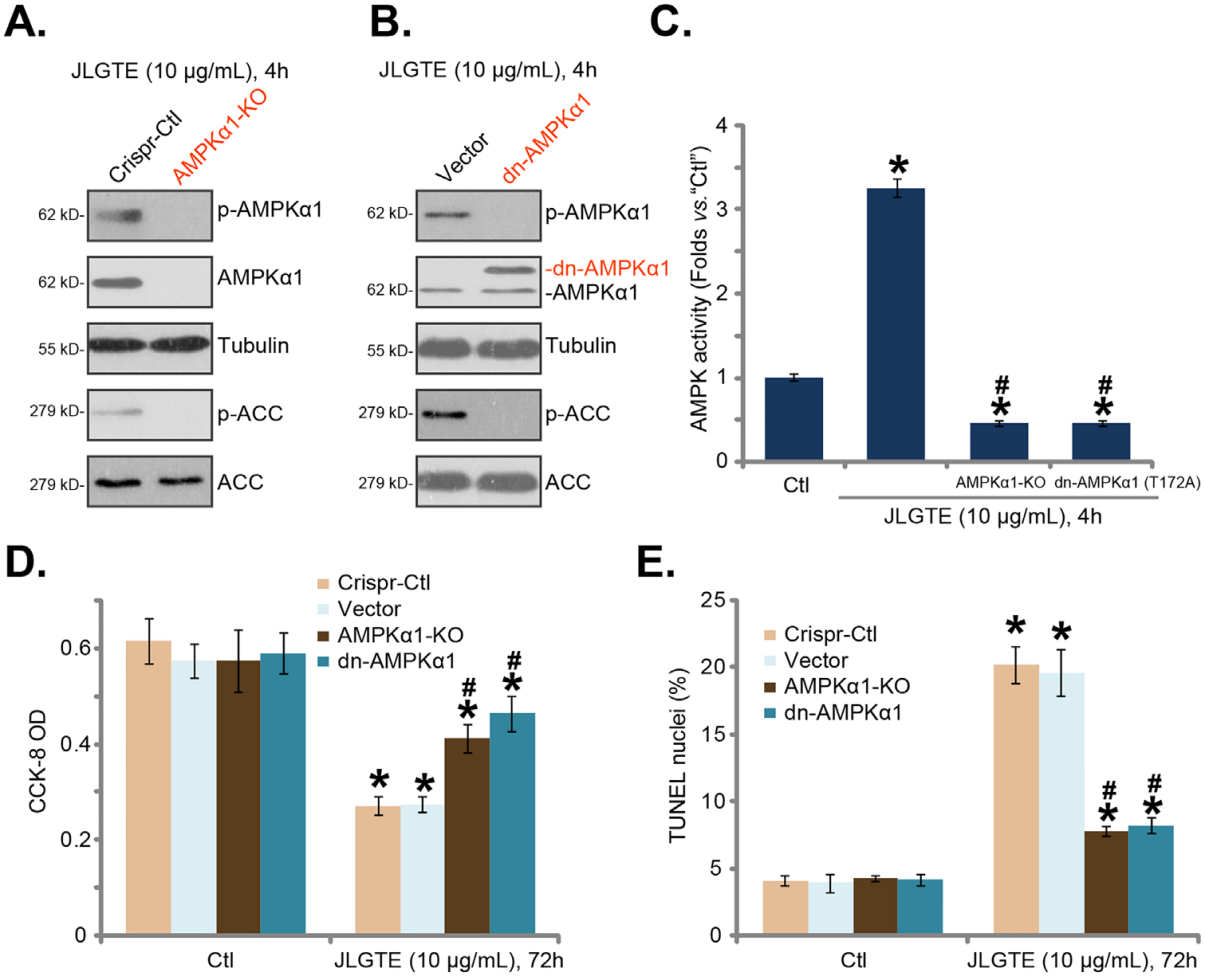
AMPKα1 knockout or dominant negative mutation attenuates JLGTE-induced cytotoxicity in HepG2 cells Stable HepG2 cells, expressing lenti-CRISPR-Cas9-AMPKα1 construct (“AMPKα1-KO”) or lenti-CRISPR-Cas9 control (“Crispr-Ctl”), as well as the dominant negative AMPKα1 (T172A, “dn-AMPKα1”, tagged with Flag) construct or the empty vector (“Vector”, pSuper-puro-GFP), were either left untreated (“Ctl”) or treated with JLGTE (10 μg/mL) for the applied time period, expressions of listed proteins in total cell lysates were shown **(A** and **B)**; AMPK activity was also tested **(C)**; cell survival (CCK-8 assay, **D**) and apoptosis (TUNEL staining assay, **E**) were examined. Data were presented as mean ± standard deviation (SD, with n=5). ^*^ ***P*** < 0.05 *vs.* “Ctl” group. ^#^ ***P*** < 0.05 *vs.* “Crispr-Ctl”/“Vector” cells. Experiments in this figure were repeated three times, and similar results were obtained.

The results indicated that AMPKα1 was required for JLGTE-induced AMPK activation, the latter mediated HCC cell apoptosis. To further support the hypothesis, CRISPR-Cas9 genome editing method was applied to silence AMPKα1. As described in the method section, the lentiviral CRISPR-Cas9-AMPKα1 vector was introduced to HepG2 cells, and stable cells were selected by puromycin. The Western blotting assay results in Figure 4A confirmed the complete knockout of AMPKα1 in the stable cells (“AMPKα1-KO” cells). Consequently, JLGTE (10 μg/Ml, 4 hours)-induced phosphorylations of AMPKα1 and ACC were also abolished (Figure 4A). To in-activate AMPK, a dominant negative AMPKα1 (T172A, “dn-AMPKα1”, a gift from Dr. Lu [8, 20]) was introduced to HepG2 cells. The results of Figure 4B demonstrated the expression of exogenous dn-AMPKα1 (Flag-tagged) in the stable HepG2 cells. Notably, the dn-AMPKα1 dramatically inhibited JLGTE (10 μg/mL, 4 hours)-induced phosphorylations of AMPKα1 and ACC in HepG2 cells (Figure 4B). The AMPK activity assay results in Figure 4C further showed that JLGTE (10 μg/mL, 4 hours)-induced AMPK activation was almost abolished in AMPKα1-KO cells or dn-AMPKα1-expressing cells. More importantly, JLGTE (10 μg/mL)-induced cytotoxicity against HepG2 cells was largely attenuated in both AMPKα1-KO HepG2 cells and dn-AMPKα1-expressing HepG2 cells (Figure 4D and 4E). These genetic evidences further confirmed that activation of AMPK is required for JLGTE-induced cytotoxicity in HCC cells.

### Forced-activation of AMPK is cytotoxic to HepG2 cells

**Figure 5:**
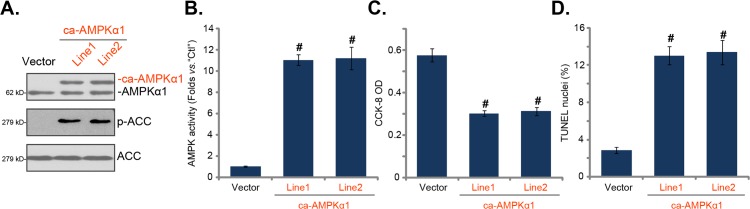
Forced-activation of AMPK is cytotoxic to HepG2 cells Stable HepG2 cells, expressing the constitutively-active AMPKα1 (“ca-AMPKα1”, T172D, two lines, “Line1/2”) or the empty vector (“Vector”, pSuper-puro-GFP), were subjected to Western blotting assay **(A)** and AMPK activity assay **(B)**; cell survival (CCK-8 assay, **C**) and apoptosis (TUNEL staining assay, **D**) were also tested. Data were presented as mean ± standard deviation (SD, with n=5). ^#^ ***P*** < 0.05 *vs.*“Vector” cells. Experiments in this figure were repeated three times, and similar results were obtained.

Based on the above results, we hypothesized that forced-activation of AMPK should mimic JLGTE's actions in HepG2 cells. To test this hypothesis, a constitutively-active AMPKα1 (“ca-AMPKα1”, T172D [37, 38]) construct was introduced to HepG2 cells. Two stable cell lines with ca-AMPKα1 were established (“Line1/2”) via selection. The Western blotting assay results in Figure 5A confirmed expression of exogenous ca-AMPKα1 (Flag-tagged) in the stable cells, where ACC phosphorylation was significantly increased (Figure 5A). The basal AMPK activity in ca-AMPKα1-expressing HepG2 cells was significantly higher than vector control HepG2 cells (Figure 5B). Remarkably, as compared to the control cells, ca-AMPKα1-expressing HepG2 cells presented with decreased cell viability (Figure 5C), but increased cell apoptosis (Figure 5D). These results showed that forced-activation of AMPK by ca-AMPKα1 exerted cytotoxicity to HepG2 cells.

## DISCUSSION

In this study, we convincingly showed that JLGTE activated AMPK signaling in both established (HepG2) and primary human HCC cells. AMPK activation was evidenced by phosphorylations of AMPK and ACC as well as increased AMPK activity in JLGTE-treated HCC cells. We suggested that AMPK activation was required for JLGTE-mediated anti-HCC cell activity. AMPKα1 silence, using targeted-shRNAs or CRISPR-Cas9 genome editing method, not only abolished JLGTE-induced AMPK activation, but also largely attenuated following HCC cell death and apoptosis. Further, in-activation of AMPK by the dn-AMPKα1 similarly decreased JLGTE's cytotoxicity against HepG2 cells. Reversely, forced-activation of AMPK by introducing the ca-AMPKα1 mimicked JLGTE's actions and induced HCC cell apoptosis. Thus, these evidences clearly supported that activation of AMPK is required for JLGTE-mediated anti-HCC cell activity. Future studies will be needed to explore the underlying mechanisms of AMPK activation by JLGTE, along with the downstream targets of AMPK in mediating JLGTE-induced HCC cell apoptosis. It would also be important to verify AMPK activation by JLGTE in other cancerous cells.

It should be noted that in-activation (by dn-AMPKα1), knockdown (by targeted shRNAs) or complete knockout (by CRISPR-Cas9 method) didn't totally abolish JLGTE's cytotoxicity in HCC cells. Further, forced-activation of AMPK by ca-AMPKα1, which even greater AMPK activation than JLGTE treatment, only induced weaker HepG2 cell death and apoptosis (*vs.* JLGTE). These results suggested that other mechanisms besides AMPK activation could also be involved in JLGTE-induced actions in HCC cells, although AMPK activation apparently was certainly important.

Currently, the clinical management of HCC remains a substantial challenge [39–41]. Although surgical resection of tumor tissues seems promising, it is only available for the well-defined and early-stage tumors. Further, a high recurrence and/or metastasis rate will further cause disease-related mortalities [39–41]. There is an extremely urgent need for improved postsurgical preventive/therapeutic clinical interventions [39–41]. The results of this study imply that it shall be interesting to further test JLGTE's activity against human HCC cells in future.

## MATERIALS AND METHODS

### Reagents

Jujube leaf green tea extracts (JLGTE) was extracted from pure jujube leaf green tea using the described method [4], and was provided by Min-de Biotech (Suzhou, China). All antibodies of this study were purchased from Cell Signaling Tech Co. (Denver MA). Z-DEVD-cho (the caspase-3 specific inhibitor) and z-VAD-cho (the pan caspase inhibitor) were purchased from Selleck (Beijing, China). The cell culture reagents were provided by Gibco BRL (Shanghai, China).

### Culture of established cell lines

HepG2 cells and the L02 human hepatocytes were purchased from the Cell Bank of Shanghai Biological Institute (Shanghai, China). Cells were cultured in RPMI 1640/DMEM with 10% FBS. Cells were routinely subjected to mycoplasma and microbial contamination examination. Population doubling time, colony forming efficiency, and morphology were checked every two months to confirm the genotype.

### Culture of primary human cells

The primary human HCC cells were derived human patients, and were provided as gifts from Dr. Zhang [29]. Briefly, the surgical-isolated fresh HCC tissues were minced and incubated with Liberase (Roche Diagnostics, Shanghai, China) for 15 min. The cell suspensions were washed, filtered through a 70-μm cell strainer, and were seeded onto collagen-coated Petri dishes. The primary cancer cells were cultured in the medium for the primary cancer cells [31]. Fibroblasts and endothelial cells were abandoned. The primary human hepatocytes were provided by Dr. Fan [31], and cells were cultured as previously described [31]. All investigations were in accordance with the principles expressed in the Declaration of Helsinki. The procedures were approved by the Ethics Review Board of all authors institutions.

### CCK-8 assay of cell survival

To test cell viability, cell counting kit-8 (CCK-8, Sigma, Shanghai) assay was performed. CCK-8 absorbance optic density (OD) at 450 nm was recorded.

### BrdU incorporation assay

The HCC cells and hepatocytes were seeded initially (3×10^3^ per well, into 96-well tissue culture plate), and were exposed to JLGTE. After the indicated time period, BrdU incorporation ELISA colorimetric assay (Roche, Indianapolis, IN) was utilize to test cell proliferation. The ELISA OD at 450 nm was recorded.

### TUNEL assay

Cell apoptosis was tested by the TUNEL staining assay. At least 300 cells per treatment in five-random views under fluorescence microscope (Zeiss, Shanghai, China, 1 to 100) were included to calculate the TUNEL-nuclei percentage [42]. Nuclei were visualized using Hoechst 33342 dye (Sigma).

### Western blotting

The cell lysis buffer (purchased from Biyuntian Co, Wuxi, China) was utilized to achieve total protein lysates. After quantification, 30 μg total lysate proteins per treatment were separated by the SDS-PAGE gels (10-12% [43, 44], which were then electrically transferred to polyvinylidene difluoride (PVDF) blots (Millipore, Shanghai, China). After blockage, the designated primary and corresponding secondary antibodies were added. We utilized enhanced chemiluminescence (ECL) reagents (Pierce, Nantong, China) to detect the signaling of the interested bands by the x-ray film [38, 45, 46]. Total gray of each band was quantified via the ImageJ software.

### Colony formation assay

After the indicated JLGTE treatment, HepG2 cells (1×10^4^ cells per dish) were mixed with 1 mL agar (0.5%, Biyuntian, Wuxi, China). Cells were placed on the top of 1 mL bottom ultra pure agar (1%) in complete medium. JLGTE-containing medium was renewed every 2 days for a total of 6 passages (12 days). Afterwards, surviving colonies were counted manually.

### Caspase-3 activity assay

The caspase-3 activity was tested through the CaspASE Assay System Colorimetric Kit (Promega, Nanjing, China). Briefly, 30 μg of total cell lysates per treatment were mixed with 30 μL caspase assay buffer, with 2 μL DMSO, 10 μL DTT, and 80 μL deionized water into, as well as the caspase-3 substrate. Afterwards, pNA absorbance was measured spectrometrically (405 nm).

### Single-stranded DNA analysis of apoptosis

We utilized the single-stranded DNA (ssDNA) Apoptosis ELISA Kit (Chemicon International, Temecula, CA) to quantify cell apoptosis, using the attached protocol. The detailed procedure was described in other studies [47, 48]. ssDNA ELISA OD at 405 nm was recorded.

### AMPK activity assay

After the treatment, total cell lysates were immunoprecipitated with anti-pan-AMPKα antibody (Santa Cruz Biotech, Shanghai, China). The AMPK activity was measured in the kinase assay buffer described [49] together with the AMP-[γ-^32^P] ATP mixture, and SAMS (HMRSAMSGLHLVKRR) peptide [49]. The reaction was terminated by spotting the reaction mixture on phosphocellulose paper (P81), and the mixture was extensively washed with 150 mM of phosphoric acid. The radioactivity was measured with scintillation counter.

### AMPKα1 mutation

The dominant negative AMPKα1 (dn-AMPKα1, T172A) and the constitutively-active AMPKα1 (“ca-AMPKα1”, T172D), both in lenti-pSuper-puro-GFP-Flag vector, were gifts from Dr. Pei-hua Lu [8, 20]. HepG2 cells were seeded onto six-well plates for 12 hours in serum free medium, the dn-AMPKα/ca-AMPKα1 (0.25 μg/mL each) was transfected to HepG2 cells by the Lipofectamine 2000 (Invitrogen, Shanghai, China). Stable cells were selected by puromycin (2.5 μg/mL, Sigma) for additional 6-8 days. The expression of exogenous mutant AMPKα1 was verified via Western blotting assay.

### AMPKα1 shRNA

A total of eight different lentiviral AMPKα1 shRNAs, targeting non-overlapping sequences, were designed by Genepharm (Shanghai, China), and were added to HepG2 cells for 24 hours. Stable cells were selected by puromycin (2.5 μg/mL, Sigma) for additional 6-8 days. The AMPKα1 knockdown in the stable cells was tested by Western blotting assay. Of the tested shRNAs, Sequence 1 (“S1”), S5 and S7 shRNAs efficiently downregulated AMPKα1 in HepG2 cells. Control cells were infected with scramble non-sense control shRNA.

### AMPKα1 knockout by CRISPR/Cas9 genome editing

Human AMPKα1's small guide RNA (sgRNA) was based on a previous study [50], which was inserted into the lenti-CRISPR plasmid (Addgene, Shanghai, China). The construct was transfected to HepG2 cells. Stable cells were selected by puromycin (2.5 μg/mL, Sigma) for additional 6-8 days. Complete AMPKα1 knockout was verified by Western blotting assay.

### Statistics

All data were presented as the mean ± standard deviation (SD) of at least three independent experiments. Statistical comparisons were performed using one-way analysis of variance (ANOVA) followed by Tukey's post hoc test for multiple comparison (SPSS 18.0, Chicago, CA). **P** values of <0.05 were considered statistically significant.
